# Retrosynthesis with attention-based NMT model and chemical analysis of “wrong” predictions†

**DOI:** 10.1039/c9ra08535a

**Published:** 2020-01-08

**Authors:** Hongliang Duan, Ling Wang, Chengyun Zhang, Lin Guo, Jianjun Li

**Affiliations:** Artificial Intelligent Aided Drug Discovery Lab, College of Pharmaceutical Sciences, Zhejiang University of Technology Hangzhou 310014 P. R. of China hduan@zjut.edu.cn lijianjun@zjut.edu.cn; Department of Pharmacy, The Affiliated Hospital of Xuzhou Medical University, Jiangsu Key Laboratory of New Drug Research and Clinical Pharmacy, Xuzhou Medical University Xuzhou Jiangsu 221000 P. R. of China

## Abstract

We consider retrosynthesis to be a machine translation problem. Accordingly, we apply an attention-based and completely data-driven model named Tensor2Tensor to a data set comprising approximately 50 000 diverse reactions extracted from the United States patent literature. The model significantly outperforms the seq2seq model (37.4%), with top-1 accuracy reaching 54.1%. We also offer a novel insight into the causes of grammatically invalid SMILES, and conduct a test in which experienced chemists select and analyze the “wrong” predictions that may be chemically plausible but differ from the ground truth. The effectiveness of our model is found to be underestimated and the “true” top-1 accuracy reaches as high as 64.6%.

## Abstract 2

We consider retrosynthesis to be a machine translation problem.

## Graphical abstract

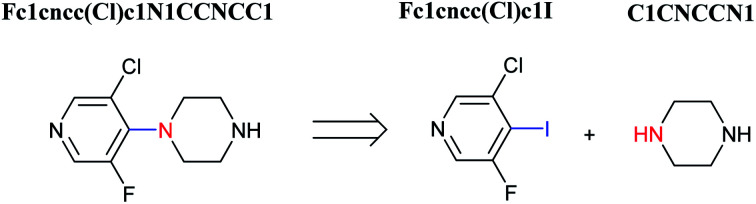

## Introduction

**Fig. 1:**
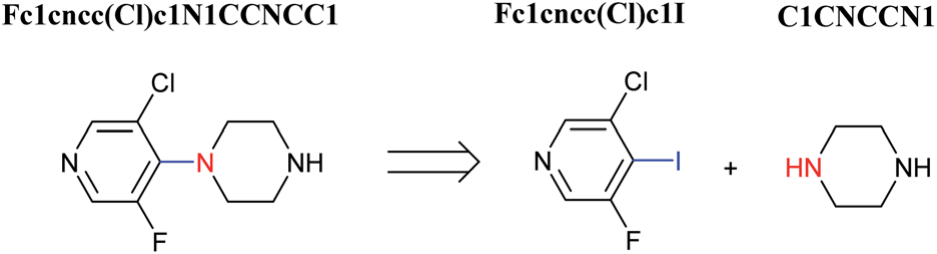
An example of a retrosynthesis reaction. The target compound is shown to the left of the arrow and potential reactants are shown to the right. SMILES representations for each are also indicated.

Organic synthesis is a crucial cornerstone of the pharmaceutical, biomedical, and materials industries. There are two closely related issues which contribute to the synthesis of new molecules: reaction prediction and retrosynthesis. The task of the former is to deduce what may be the underlying product of a given set of reaction building blocks such as reactants, reagents, and reaction conditions. For retrosynthesis, the problem is approached in reverse: starting from the target compound chemist hunger for creating, and exploring the simpler precursors commercially available (Fig. 1).^1–3^ By sequentially combining all of the reactions derived from a retrosynthetic analysis, an overall synthetic route to a target molecule can be identified.

Over the past few decades, various methods involving novel and emerging computing techniques have been developed to perform retrosynthetic analysis.^4–6^ Since the 1960s, computers have been used to store chemical structures data, and to apply chemical structural information to synthesis planning and drug discovery.^7^ The first retrosynthesis program created by Corey and Wipke introduced computer assistance to chemical synthesis. This program called Logic and Heuristics Applied to Synthetic Analysis (LHASA).^8^ They pioneered the use of expertly crafted rules which are regularly alluded to reaction templates.

Another approach to retrosynthesis is Knowledge base-Oriented system for Synthesis Planning (KOSP).^9^ The system was built on the knowledge base in which reactions were abstracted according to the structural characteristics of reaction sites and their environments. Generally, computer-assisted retrosynthetic analysis is performed by exploiting reaction rules which include a series of tiny transformations to characterize chemical reactions. These rules can either be laboriously encoded by chemical experts, or extracted from various sources of chemical digital data.^10–18^ The outstanding advantage of the rules is that they can be interpreted directly. However, the rule-based methods remain several drawbacks. First, since there currently is no comprehensive rule system which cover all chemical fields, rule-based systems cannot synthesize new compounds with methods outside the current knowledge base. In addition, the rules need to be coded and curated, and this is prohibitively expensive and time consuming.

Deep learning (DL) is a class of machine learning algorithms which are intended to teach an artificial neural network (ANN) containing a multi-layer nonlinear processing unit data representations.^19^ Since the earliest ANN was established in 1943, significant improvements have been made between the 1960s and 1980s.^20^ Moreover, recent advances in DL for computer games and self-driving cars have demonstrated the wide-ranging potential applications of DL.^21^

Given the increased availability of a wide variety of digital data and algorithms, DL represents a valuable resource for managing reaction data for retrosynthetic analysis. Recently, data-driven approaches have been employed to circumvent the restrictions of rule-based systems. For example, molecules can be equivalently represented as text sequences, as demonstrated with the simplified molecular-input line-entry system (SMILES).^22^ From a linguistic perspective, this system can be regarded as a language, and a chemical reaction can be treated as a translation task. Nam and Kim were the first to introduce sequence-to-sequence (seq2seq) model which is a neural machine translation (NMT) model to reaction prediction. They mapped the SMILES representations of reactants to the SMILES representations of products.^23^ Subsequently, Schwaller *et al.* further built on the idea of relating reaction prediction to a language and explored the potential of an NMT method known as the seq2seq model.^24^

Given that retrosynthesis is the opposite of reaction prediction, the hypothesis to be tested is that the seq2seq model could deal with retrosynthesis problems as reaction prediction in reverse. Liu *et al.* tested this hypothesis by formulating retrosynthesis as translation task for the seq2seq model.^25^ This was achieved by establishing a SMILES target compound, then receiving a SMILES reactant output. When using this approach, 37.4% accuracy was achieved for top-1, and 52.4% and 61.7% accuracies were achieved for top-3 and top-10, respectively. Thus, this approach performed comparably or worse than a rule-based expert baseline model. Meanwhile, the grammatical invalidity rate of the top-1 predicted SMILES was greater than 10%. Therefore, the potential application of this program to future retrosynthetic reaction prediction is limited.

Herein, we present an attention-based NMT model, Tensor2Tensor (T2T) model, which exhibits great superiority to the machine translation tasks while being more parallelizable and requiring significantly less time to train.^26^ Similar approaches have recently been suggested.^27,28^ In this paper, our team focus on a central challenge of retrosynthesis: “Given a target product, what are the most likely reactants?” The innovative T2T model we described is applied to retrosynthesis, which procure higher top-1 accuracy (54.1%) than previous work^25^ and 3% invalidity of the top-1 predicted reactants SMILES on a common benchmark database. Diverse parameters such as batch size and training time are investigated to train the model. We find that batch size should be set as high as possible, while keeping a reserve for not hitting the out-of-memory errors. Meanwhile, extending even training time has the potential to yield better performance. In addition, we analyse incorrect SMILES results and discover that two factors, complexity of chemical structure and a lack of training data, may lead to failure in text presentation. Finally, we conduct a test in which ten experienced chemists pick out and analyse “wrong” predictions that may be true from chemists' point of view but inconsistent with the ground truth.

## Results

### Data

We adopt the same dataset of reactions as in Liu's work.^25^ The dataset containing reaction examples derived from United States Patent and Trademark Office (USPTO) patents was originally prepared by Lowe.^29^ Schneider *et al.* extracted approximately 50 000 reactions spanning 10 broad reaction types (Table S1†) from it to represent the typical reaction types found in the medical chemists' toolkit and discarded contextual information (*e.g.*, temperature, reagents, yields) for only comprising reactants and products.^30^ Additionally, Liu *et al.* further developed this dataset by splitting multiple product reactions so that each reaction example contains a single product. Finally, there are 40 029 reactions for training, 5004 reactions for validation, 5004 reactions for testing, and all reactions are described by a text-based representation called SMILES.

### T2T model

T2T model is implemented to retrosynthetic reaction prediction. This model based on an encoder–decoder architecture initially is constructed for NMT tasks^31^ and it shows state of the art performance in chemical reaction prediction.^32^

The architectural characteristic of T2T model is that it entirely depends on attention mechanisms. As a new generation of encoder–decoder neural network model, T2T model, comprising feed-forward network and multi self-attention layers, avoids complicated recurrent or convolutional neural networks. It can get queries (*Q*) that data inquires, search keys (*K*) for the indexed knowledge and acquire values (*V*) related to queries and keys, then matrixes learn them during training. In order to obtain queries (*q*), keys (*k*), values (*v*) corresponding to a current batch, the T2T model multiply *Q*, *K*, *V* with the input (*X*). With these computed parameters, the inputs can be transformed to some encoding parts or decoding parts.^26^

**Fig. 2:**
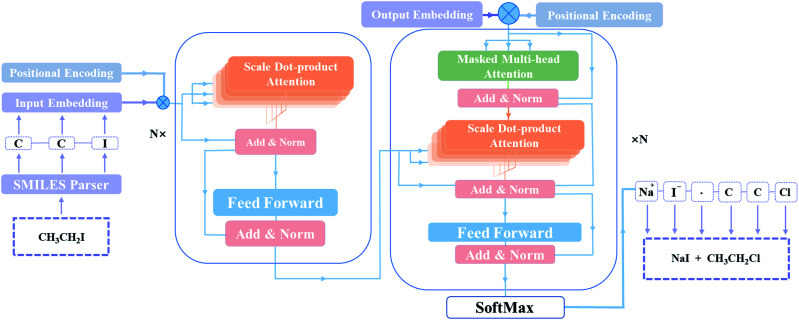
The T2T model architecture. Inputs and outputs are described by SMILES in this model. The encoder (left) and the decoder (right) consist of a stack of *N* identical layers. *N* is the number of layers.

As depicted in Fig. 2, the main components of the model are encoder and decoder stacks. The encoder is composed of several same layers and each layer contains two different sub-layers. The first sub-layer is a multi-head self-attention mechanism and the second is a feed-forward network layer. Before layer normalization,^33^ a residual connection^34^ is applied around each of the two sub-layers. The decoder consists of identical layers but each layer is comprised of three different sub-layers. Apart from the two sub-layers mentioned, there is a third sub-layer called masked multi-head self-attention mechanism, and the residual connection is still employed around each of the sub-layers as well as the encoder.

Remarkably, the multi-head attention which consists of parallel attention layers is an innovative part of T2T model. We can perform the attention function in parallel to get different versions of output values after linearly projecting the queries, keys, and values, then concatenate and again project them to obtain the final values. Hence, the model with several sets of independent attention parameters outperforms models with a single attention function. Several scaled dot-product attention layers make up a multi-head attention layer. They take the input made up of queries, keys of dimension dk, and values of dimension dk, then calculate the three entities. The dot products of the queries are computed with all keys to explore alignment between the keys with them, then we multiply the results by 
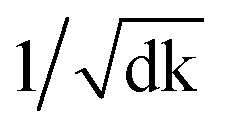
, and get the weights of the values. The queries, keys, and values that the attention computes will be packed together into matrices in practice so that we can get a matrix of outputs.

T2T model pays same attention to the elements of sequences no matter how long distances between tokens, resulting in information about the relative or absolute position of tokens in sequences may be missing. A positional encoding matrix is proposed to solve such problems. Depending on the positions in sequences and in the embedding directions, the elements equal to the values of trigonometric functions. The positional encoding make connection between far located parts of the inputs with learned embedding. With it, the model can make use of the order of the sequences.

### Baseline model

Baseline model is seq2seq model^35^ which was employed by Liu *et al.* to perform retrosynthesis. The primary parts of the model are the encoder and decoder parts which are both made up of long short-term memory (LSTM) cells,^36^ a variant of recurrent neural network cells. In the architecture, the encoder takes sequences of the inputs and trains them then passes corresponding context vector to the decoder, and the decoder uses the representation and gives sequences of the outputs. However, there are some problems in this model. One major drawback of seq2seq model is that with recurrence operation, the computation cannot be parallelized on multi GPUs. In addition, the main limitation in the architecture's ability to process sequences is the size of information the fixed-length encoded feature vector can contain. And the performance of the model decreases as the length of sequences increases. Therefore, it is rather challenging for seq2seq model to tackle too long sequences.

The seq2seq model is based on TensorFlow (version 1.01), which all scripts are written in Python 3.5 and the T2T model is built with TensorFlow (version 1.11.0),^37^ which all program scripts are written in Python 2.7. The open-source cheminformatics toolkit RDKit^38^ (version 2019.03.10) is applied for analysing the reaction data.

### Comparison of accuracies between baseline and T2T models

**Fig. 3:**
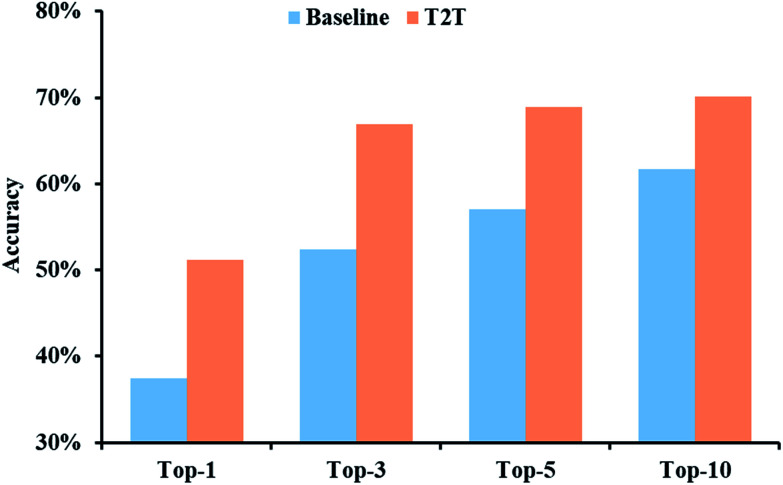
Top-*N* accuracies of the baseline and T2T models for a test data set.

The prediction accuracies of baseline and T2T models in regard to top-*N* are described in Fig. 3. Our model exhibits greater superiority to the baseline model on top-1 accuracy by a large margin. Moreover, with an increase in *N*, a prominent improvement in model accuracy is further observed. For example, the top-10 accuracy of the T2T model reaches 70.1%, while that of the baseline model reached 61.7%.

**Fig. 4:**
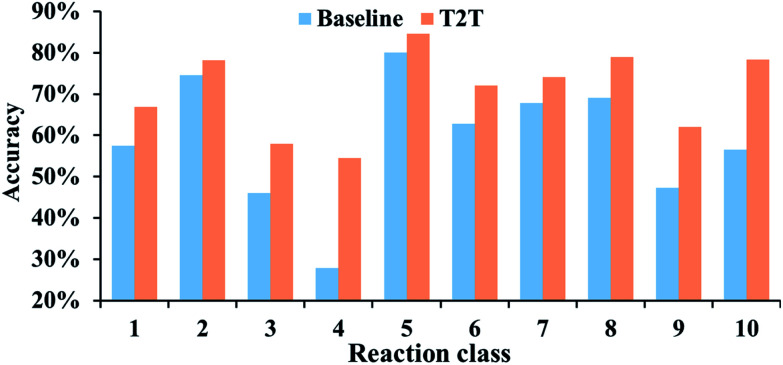
Top-10 accuracies of the baseline and T2T models for the various reaction classes.

The performances of our model for different reaction types are also explored and we compare the results to the baseline model. For all of the reaction types examined, our model performs significantly better (Fig. 4). Take reaction class 3 as an example, the accuracy of C–C bond formation reactions in our model achieves 58.0%, which is 11.9% higher than the result (46.1%) achieved with the baseline model.

**Fig. 5:**
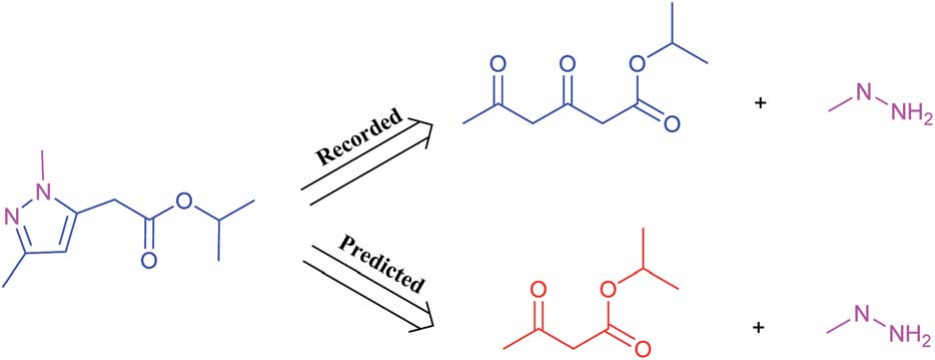
A representative example of a recorded *versus* wrong predicted cycle formation reaction of T2T model.

It is worth noting that reaction class 4, which includes the formation of cyclic structure, usually leads to great variations between reactants and products (Fig. 5). Thus, even for an experienced chemist, it is a challenging aspect of retrosynthesis to decide the proper disconnection bond for a ring. The T2T model achieves an accuracy level of 54.4% for reaction class 4, which markedly exceeds the accuracy achieved with the baseline model (27.8%). However, this result is not as good as that achieved for the other reaction types, and this is attributed to the complexity of cyclic structures.

### Effect of batch size

Batch size is the number of training samples processed in one training step and it is often a critical parameter of a model.^39^ For example, batch size has been shown to affect prediction quality, training speed, and training stability in other neural network architectures.^40^ In our model, the parameter, batch size, is defined as the number of tokens in a batch. To evaluate the influence of this parameter on our attention-based model, we perform a set of experiments with batch size varying from 512 to 8192.

**Fig. 6:**
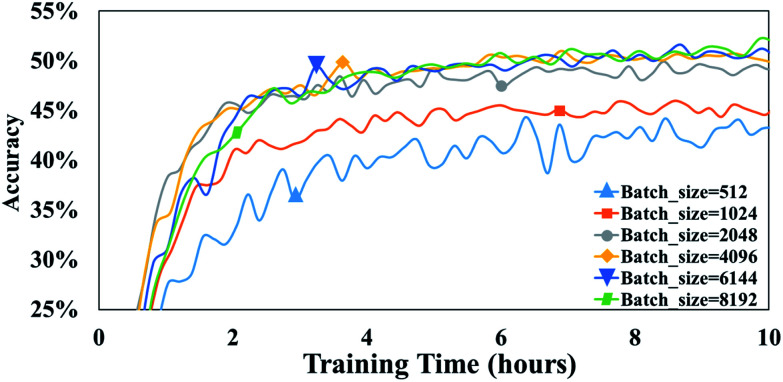
The accuracies of T2T model with different batch sizes. Note that all are trained and tested on a 1080Ti GPU.

After 10 h of training, 43.4% accuracy is achieved with a batch size of 512, and 49.2% accuracy is achieved with a batch size of 2048 (Fig. 6). Thus, it appears that larger batch sizes perform better than smaller ones. However, the accuracy does not significantly increase any more when the batch size exceeds 2048. For example, after 10 h of training, an accuracy of 50.1% accuracy is achieved with a batch size of 4096 is compared with 50.6% accuracy with a batch size of 6144. Furthermore, there is no substantial difference between the accuracies achieved with batch sizes of 4096 and 6144.

A possible explanation of this phenomenon is that training throughput which is the number of training data processed in the training, markedly affects the performance of the T2T model when a high batch size is involved. To our knowledge, the bigger the batch size is, the slower computation speed is, and as batch size becomes larger, training throughput which equals to multiply batch size by computation speed improves slightly.^39^ Consequently, the predictive capabilities do not greatly increase when batch size exceeds a certain size, as a result of a mildly higher throughput. Note that setting a batch size too high may result in out-of-memory errors. Conversely, setting a batch size too low could lead to notably low accuracies. Thus, it is of a great advantage to employ proper batch size.

### Influence of training time

In our experiments, accuracies do not increase markedly after several hours of training. To explore whether a more extended training time could provide better result, a test with a training time as long as 5 day on our GPU is carried out.

As illustrated in Table 1, a batch size of 8192 achieves an accuracy of 51.8% after 10 h of training. This accuracy is further improved to 53.0% after 5 days of training. Taken together, these results indicate that our model can potentially achieve marginally better result with longer training time. We conduct an additional experiment by averaging checkpoints to improve accuracy. By averaging the last 20 checkpoints saved in 2000-step intervals and training the model for 5 days, we achieve an accuracy of 54.1% with a batch size of 8192. Compared to previous work, using of checkpoint averaging in the present study results in a higher probability of correct predictions. Thus, it is advised that checkpoint averaging be applied to our model.

**Table 1:** Influence of batch size and training time on performance of the T2T model

Batch size	Training time	Accuracy (%)
512	10 h	43.4
1024	10 h	45.7
2048	10 h	49.2
4096	10 h	50.1
6144	10 h	50.6
8192	10 h	51.8
8192	5 d	53.0
8192 (avg.)	5 d	54.1

## Discussion

### Grammatically invalid SMILES comparison between baseline and T2T models

The invalidity rate of the top-1 predicted reactant SMILES strings in the T2T model is 3.4%. In comparison, the invalidity rate was 12.2% in the seq2seq model. Liu *et al.* attached great importance to reaction types in consideration what factors cause the seq2seq model to make a lot of grammatical mistakes in the SMILES predictions.^25^ The results of the present study contradict those of Liu *et al.*, with the validity rate of SMILES being comparable for each reaction class (Fig. S1†). Thus, we do not find reaction types to be related to SMILES invalidation.

All of the compounds predicted to be grammatically invalid SMILES of seq2seq and T2T models are analysed. Two key factors which may cause models to incorrectly predict text representation are complexity of chemical structure and a lack of training data.

**Fig. 7:**
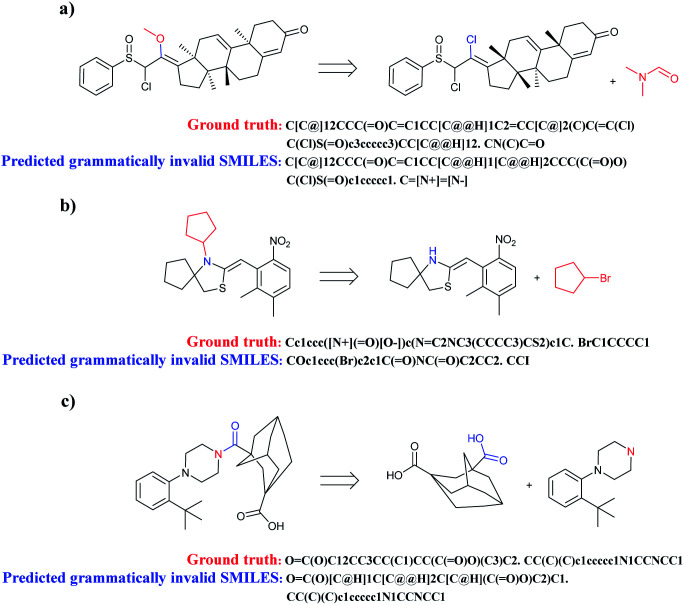
Three representative examples of cyclic compounds which are prone to be predicted to grammatically invalid reactants SMILES. (a) Polycyclic aromatic hydrocarbons; (b) spirocyclic hydrocarbons; (c) bridged hydrocarbons.

When complicated cyclic compounds such as polycyclic, spirocyclic, and bridged hydrocarbons are components of retrosynthetic analysis, seq2seq and T2T models generally output invalid SMILES (Fig. 7). A key feature of these cyclic compounds is their perplexing ring structure unit. Correspondingly, it is challenging for chemists to name these compounds. Taking spirocyclic hydrocarbon as an example, the systematic naming of it based on rules is rather tough due to its excessive complexity. Moreover, a lack of relative reaction examples can also lead to wrongly predicted reactants SMILES.

In addition, quaternary carbon structures have tremendous influence on the performance of the seq2seq model. While predicting valid SMILES reactants for molecules containing Boc, CF_3_, and *t*Bu is tough for the seq2seq model, it is a trivial problem for the T2T model.

**Fig. 8:**
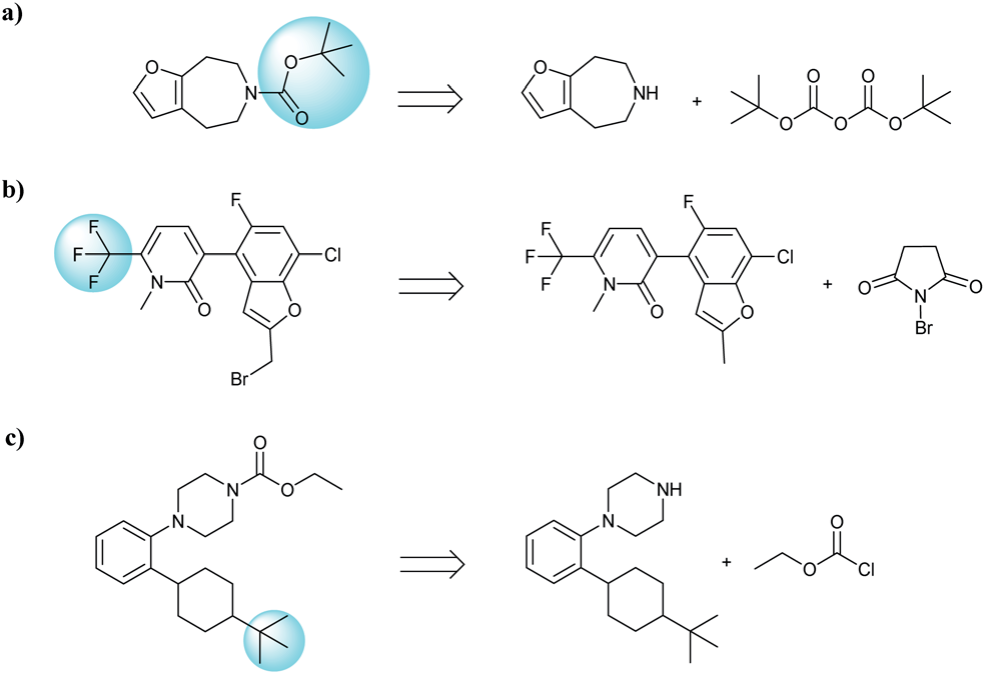
Characteristic examples of the compound containing Boc, CF_3_ and *t*Bu, which the T2T model is able to predict valid reactant SMILES, yet the seq2seq model fails.

As shown in Table 2, there are 71 predictions which containing the structures mentioned above and they account for 11.6% of the total invalid SMILES strings in the seq2seq model. In contrast, the T2T model generally does not wrongly predict this class of compounds. Fig. 8 shows representative examples for which the T2T model is capable of predicting SMILES reactants correctly, whereas for the same examples the seq2seq model fails.

**Table 2:** The distribution of wrong predictions about molecules containing quaternary carbon structures (Boc, CF_3_, and *t*Bu) in the seq2seq model

Structures	Count	Rate (%)
R-Boc	38	6.2
R-CF_3_	18	2.9
R-*t*Bu	15	2.5
Total	71	11.6

### Chemical analysis of “wrong” predictions

The accuracy of a model refers to the proportion of ground truth reactants is found in the predictions predicted by the model. However, in the present study, we further consider that a lot of chemically plausible answers which have been proven to be feasible, or those which have not been discovered, are judged to be “wrong” answers. When this happens, the accuracy of a model is reduced. To quantitatively appraise this, we conduct a test in which contains ten chemists with rich experience in organic chemistry are asked to identify “wrong” answers from sets of reactions they were provided. The participating chemists are allowed to resort to repositories such as SciFinder^41^ and Reaxys,^42^ which are web-based tools for retrieval of chemistry information and data from published literature. Representative examples of the “wrong” predictions are shown in Table 3.

**Table 3:** Examples of chemically plausible predictions considered to be “wrong” answers. (a) Hydrolysis from varied carboxylic esters to acids; (b) oxidation by different oxidants; (c) protection with diverse protecting groups; (d) C–C bond formation *via* cross-coupling reactions; (e) S_N_2 between alkoxides/amines and alkyl halides

Reaction ID	Targets	Ground truth	T2T model top-1 predictions
a	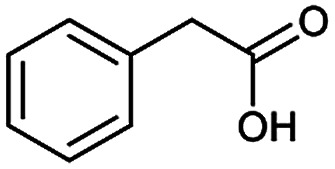	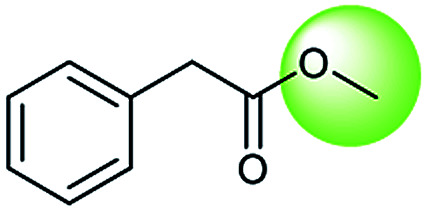	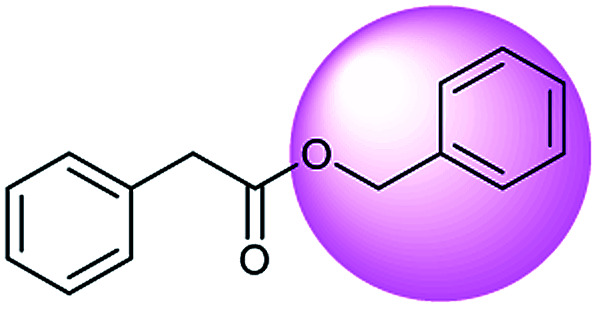
b	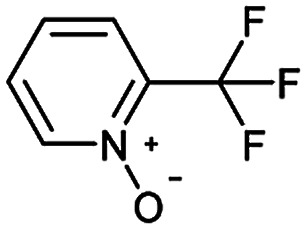	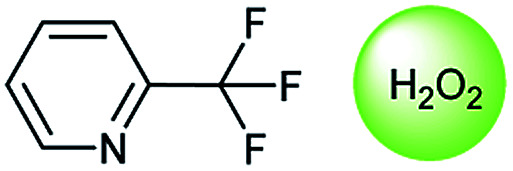	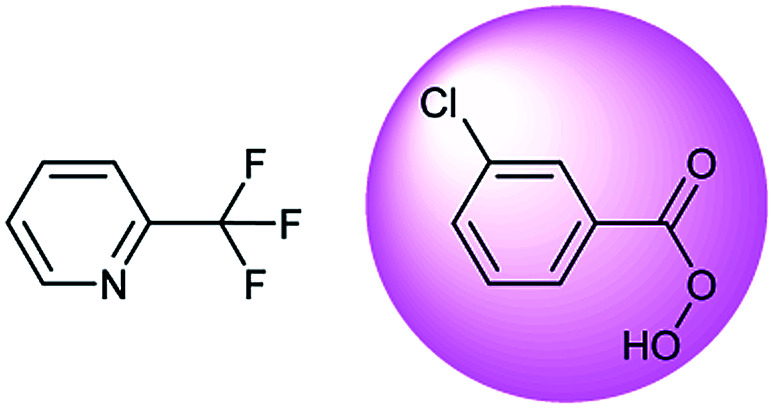
c	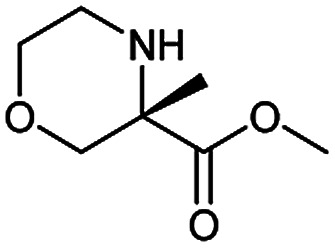	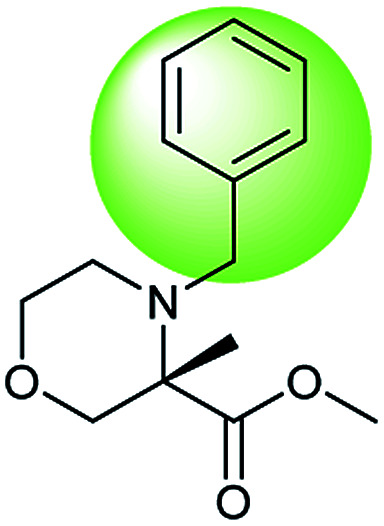	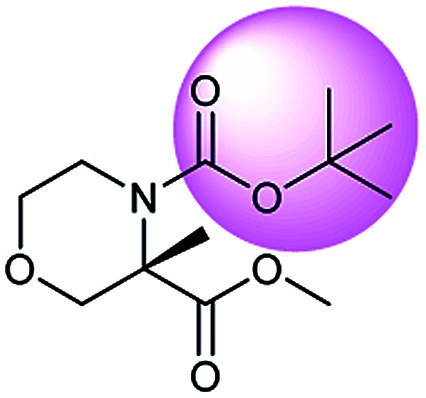
d	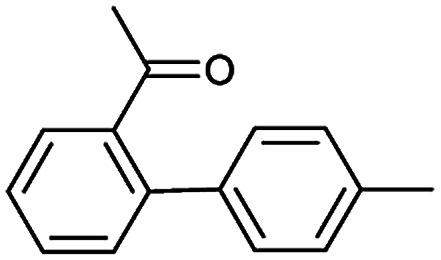	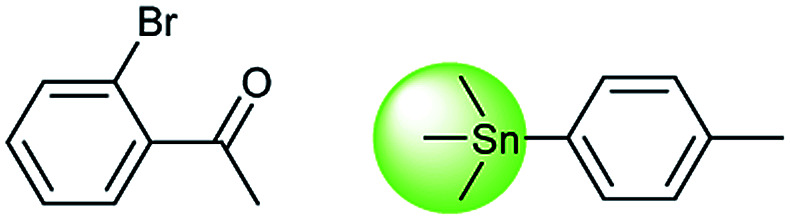	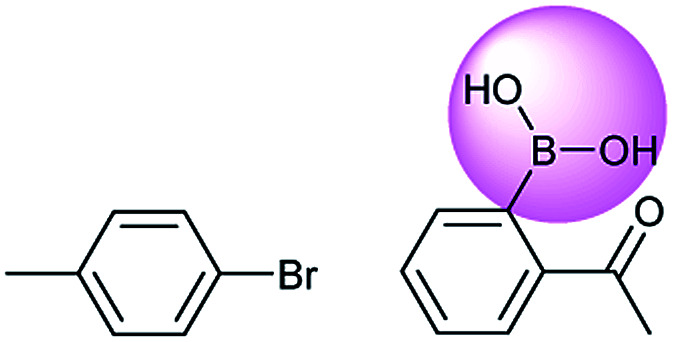
e	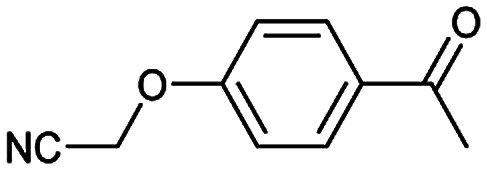	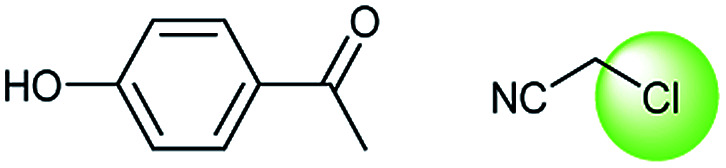	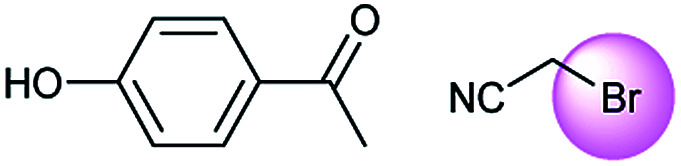

**Fig. 9:**
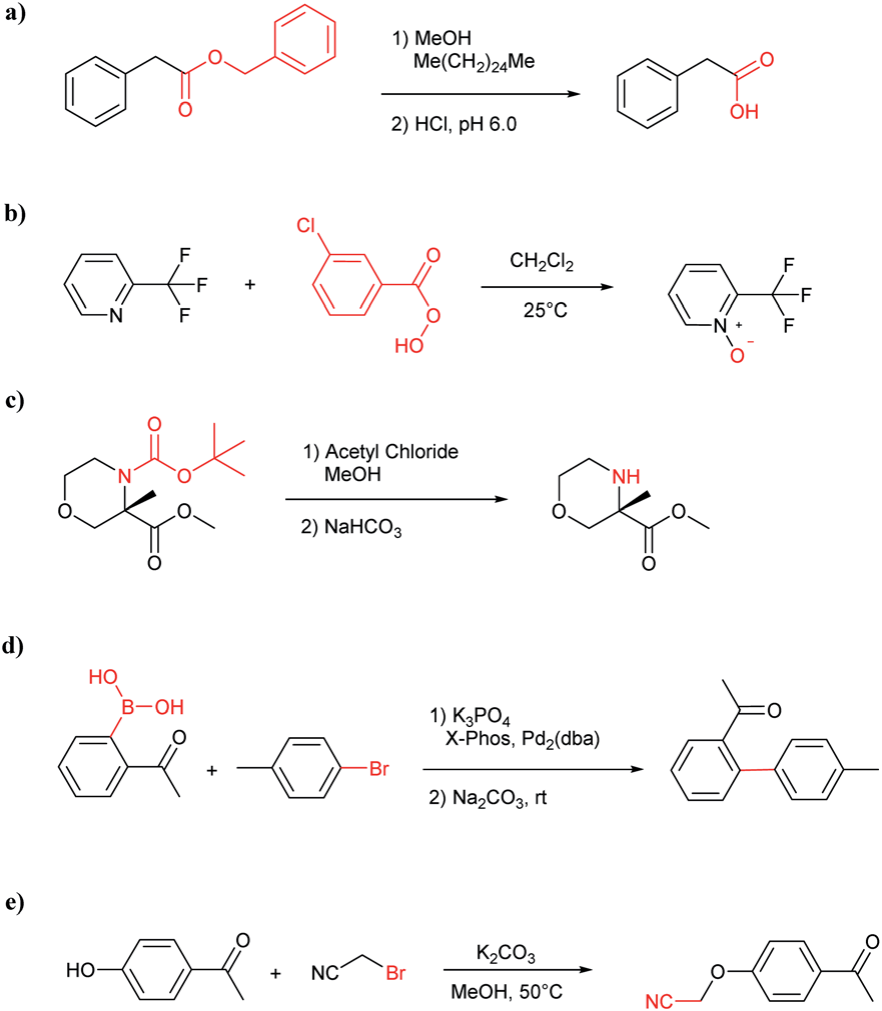
Examples of reactions predicted to be “wrong” yet which match published routes. (a) Hydrolysis of a benzyl ester; (b) oxidation *via* mCPBA; (c) protection with Boc; (d) C–C bond formation *via* Suzuki reaction; (e) a S_N_2 etherification reaction between alkoxide and bromoalkene.

The simplest method for synthesizing an acid involves hydrolysis of a carboxylic ester. For example, both benzoic acid methyl ester and benzoic acid phenylmethyl ester can form benzoic acid *via* hydrolysis.^43^ As shown in Table 3 (a), the recorded outcome shows that the target compound can be synthesized from hydrolysis of the corresponding methyl ester. The prediction is consistent with the method of Leggio *et al.*^44^ (Fig. 9a), which employed hydrolysis of the corresponding benzyl ester to produce the target compound.

Oxidation is a large class of chemical reactions in which the atoms of an element lose an electron. In Table 3 (b) illustrates retrosynthetic analysis of 2-(trifluoromethyl) pyridine which is the key intermediate of BRaf inhibitor. There are a number of oxidants which contain electrophilic oxygen atoms, and these can react with nucleophilic pyridines to produce the target compound. The most commonly used oxidation agents are *meta*-chloroperoxybenzoic acid (*m*CPBA)^45^ and H_2_O_2_ which are actually comparable to each other in this reaction. The prediction is chemically plausible due to the oxidizing agent, mCPBA, although it is likely missed in our recorded reaction examples. Aquila *et al.*^46^ have reported an approach for obtaining the target compound with the mCPBA (Fig. 9b), and this represents an important contribution to the synthesis of BRaf inhibitor.

During the preparation of complex organic molecules, there are often stages in which one or more protecting groups are introduced into a molecule in order to protect the functional groups from reacting. Often, these protecting groups are not removed until the reaction is completed. The first stage of this reaction is protection, and the second stage is deprotection. Synthesizing the target molecule is a stumbling block on the way to synthesize the intermediate of TORC1/2 inhibitor. Table 3 (c) describes a reaction example in which the precursor is deprotected to generate the target compound. In addition to a benzyl group in the recorded outcome, other alternative protecting groups, such as Boc, Cbz, Fmoc, *etc.*, can be applied to protect the amino group from powerful electrophiles. The prediction involving use of Boc protecting group is not captured in our recorded example, which is mistakenly considered to be a wrong answer. However, this approach has been confirmed to be chemically plausible by Hicks *et al.*^47^ (Fig. 9c).

Metal-catalyzed cross-coupling reactions, including Stille coupling and Suzuki coupling reactions, are commonly used to form a C–C bond. Consequently, these reactions play a significant role in organic synthesis. For instance, the recorded outcome showed that the target compound can be formed by Stille coupling between trimethyl(4-methylphenyl)stannane and 1-acetyl-2-bromobenzene (Table 3 (d)). Stille coupling is a versatile C–C bond forming reaction between stannanes and halides, and it has very few limitations regarding the R-groups. Meanwhile, the predicted outcome displays that the target compound can be synthesized *via* a Suzuki reaction between 1-bromo-4-methylbenzene and (2-acetylphenyl) boronic acid. This is also the route that was chosen by Laha *et al.*^48^ (Fig. 9d). These above two methods can substitute for each other on a large scale and they have profoundly affected the protocols for the construction of chemical molecules.

A universal method for generating ethers is to treat alkoxide anions with halohydrocarbons, including chloroalkane, bromoalkene and iodoalkane. It is a remarkable fact that the target molecule is the pivotal intermediate of bazedoxifene acetate used as drug for treatment or prevention of postmenopausal women osteoporosis. Since the two halohydrocarbons can be replaced mutually in S_N_2 reaction, there is no intrinsic difference between the recorded reaction and the predicted reaction. The ground truth shows a simple S_N_2 etherification reaction where 1-(4-hydroxyphenyl)ethanone reacts with 2-chloroacetonitrile to form the target compound and the prediction also displays a similar reaction which matches the published route^49^ (Table 3 (e) and Fig. 9e).

Due to space constraints, we will not describe all of the chemically plausible reaction types in detail. Furthermore, statistical measures are more intuitive than explaining the reaction examples individually. The numbers of various reaction types are indicated in Table 4. It is worth noting that examples of hydrolysis from varied carboxylic esters to acids represent the largest of the seven types of reactions. There are two additional types of reactions, condensation between carboxylic acids and amines and reductions from carbonyl compounds to alcohols, which also warrant mention, with percentages of 2.3% and 1.4%, respectively.

**Table 4:** Breakdown of chemically plausible predictions for different reaction types

Chemically plausible reaction type	Count	Rate (%)
Oxidation by different oxidants	11	0.2
Protection with diverse protecting groups	50	1.0
Hydrolysis from varied carboxylic esters to acids	127	2.5
C–C bond formation *via* cross-coupling reactions	36	0.7
S_N_2 between alkoxides/amines and alkyl halides	123	2.4
Reductions from carbonyl compounds to alcohols	69	1.4
Condensation between carboxylic acids and amines	120	2.3
Total	536	10.5

## Conclusions

In this work, we present a completely data-driven and solely attention-based T2T model for retrosynthesis. This model outperforms the seq2seq model by a large margin in every class and it exhibits important advantages over both conventional rule-based systems and any DL approaches. This indicates that our approach clearly enhances the performance of DL for retrosynthetic reaction prediction task relative to all-rules machine learning models. However, a critical limitation regarding this accuracy metric is that a large number of chemically plausible predictions are judged as “wrong” answers, and this results in a lower accuracy rate for a model compared with its “true” accuracy. For example, in the present study, 536 “wrong” predictions accounted for 10.5% of the total test data set. As a result, the “true” accuracy of the T2T model is found to reach 64.6%. The inability of the accuracy metric to match our model highlights an important limitation of all current models. One way to alleviate this issue should be put on the agenda of researchers.

In conclusion, the T2T model is a valuable resource for predicting reactions and performing retrosynthesis. However, the T2T model is originally constructed for a language translation mission and is not fully adapted to the task of retrosynthetic reaction prediction. It is believed that in the future, some slight adjustments for this model architecture can better solve the problem of retrosynthesis. While few researchers are currently engaged in this area of research, we anticipate a dramatic increase in the coming years as the practical challenges are addressed.

## Associated content

The T2T model, processed data sets, and evaluation code will be available at https://github.com/hongliangduan/RetroSynthesisT2T.git. The seq2seq model, processed data sets and evaluation code will be made accessible at https://github.com/pandegroup/reaction_prediction_seq2seq.git.^25^

## Author contributions

Hongliang Duan, Ling Wang and Chengyun Zhang contribute equally.

## Conflicts of interest

There are no conflicts to declare.

## Supplementary Material

RA-010-C9RA08535A-s001
