# Supplementary material for: Retrosynthesis with attention-based NMT model and chemical analysis of “wrong” predictions
Source: RSC Adv. 2020 Jan 8;10(3):1371–8. doi: 10.1039/c9ra08535a (PMC9047528; doi:10.1039/c9ra08535a)
Supplement: RA-010-C9RA08535A-s001 [file RA-010-C9RA08535A-s001.pdf]

Electronic Supplementary Material (ESI) for RSC Advances.
This journal is © The Royal Society of Chemistry 2019

Supporting Information

Retrosynthesis with Attention-Based NMT Model and Chemical Analysis of "Wrong" Predictions

Hongliang Duan,*[a] Ling Wang,[a] Chengyun Zhang,[a] Lin Guo[b] and Jianjun Li*[a]

[a]*Artificial Intelligent Aided Drug Discovery Lab, College of Pharmaceutical Sciences, Zhejiang University of Technology, Hangzhou 310014, P. R. of China.*

[b]*Department of Pharmacy, The Affiliated Hospital of Xuzhou Medical University, Jiangsu Key Laboratory of New Drug Research and Clinical Pharmacy, Xuzhou Medical University, Xuzhou, Jiangsu, 221000, P.R. of China.*

**E-mail:* hduan@zjut.edu.cn and lijianjun@zjut.edu.cn

Table S1 Distribution of major reaction classes with the processed reaction data set

Reaction class	Reaction name
1	heteroatom alkylation and arylation
2	acylation and related processes
3	C-C bond formation
4	heterocycle formation
5	protections
6	deprotections
7	reductions
8	oxidations
9	functional group interconversion (FGI)
10	functional group addition (FGA)

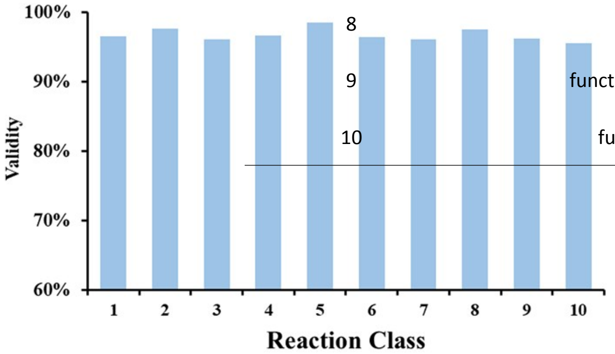

Fig. S1 Validity of SMILES in each reaction class.
